# Supplementary material for: Identification of novel non-myelin biomarkers in multiple sclerosis using an improved phage-display approach
Source: PLoS One. 2019 Dec 5;14(12):e0226162. doi: 10.1371/journal.pone.0226162 (PMC6894809; doi:10.1371/journal.pone.0226162)
Supplement: S1 Table — BlastN analysis of 15 randomly picked clones from the ORF-selected phage display cDNA library from human brain. The portion of each identified nucleotide sequence and the percentage of homology are indicated. The sequences of clones that were also coding for the corresponding peptide (“in frame” clones, IF) are indicated; the “out-of-frame” clones (OF) were non coding sequences. (PDF) [file pone.0226162.s001.pdf]

S1 Table. Sequence analysis of randomly chosen clones from HB library.

Clone	Identity(blastN)	Nt sequence	Homology	ORF
HB1	Homo sapiens neurofilament 3 (150kDa medium) (NEF3), mRNA	2315-2676	99%	IF
HB2	Homo sapiens ribosomal protein SA (RPSA), transcript variant 1, mRNA	107-418	99%	IF
HB3	Homo sapiens chromosome 5 genomic contig	42251786-42251655	100%	IF
HB4	Homo sapiens cold shock domain containing C2, RNA binding (CSDC2), mRNA	10-62	98%	OF
HB5	Homo sapiens chromosome 2 genomic contig	75938720-75938686	100%	IF
HB6	Human DNA sequence clone RP11-378J18 on chromosome 1	4894-4807	98%	IF
HB7	Homo sapiens brain expressed, X-linked 1 (BEX1), mRNA	232-489	100%	IF
HB8	Homo sapiens polymerase (RNA) I polypeptide D, 16kDa (POLR1D), transcript variant 2, mRNA	101-370	100%	IF
HB9	Homo sapiens enolase 2 (gamma, neuronal) (ENO2), mRNA	2010-1859	99%	IF
HB10	Homo sapiens microtubule-associated protein 1B (MAP1B), transcript variant 2, mRNA	1808-2034	100%	IF
HB11	Fas apoptotic inhibitory molecule 2 (FAIM2)	3615-3545	98%	IF
HB12	Vesicle-associated membrane protein 1 (VAMP1)	2038-1806	100%	IF
HB13	Transient receptor potential cation channel, subfamily V, member 6	1487-1604	100%	IF
HB14	GNAS complex locus (GNAS)	173-414	100%	OF
HB15	Chromatin assembly factor 1 (p150) (CHAF1A)	1275_1428	100%	IF

BlastN analysis of 15 randomly picked clones from the ORF-selected phage display cDNA library from human brain. The portion of each identified nucleotide sequence and the percentage of homology are indicated. The sequences of clones that were also coding for the corresponding peptide (“in frame” clones, IF) are indicated; the “out-of-frame” clones (OF) were non coding sequences.
