# Supplementary material for: Identification of novel non-myelin biomarkers in multiple sclerosis using an improved phage-display approach
Source: PLoS One. 2019 Dec 5;14(12):e0226162. doi: 10.1371/journal.pone.0226162 (PMC6894809; doi:10.1371/journal.pone.0226162)
Supplement: S2 Table — List of antigens identified by the selection of phage display cDNA library from human brain with anti-human IgG. (PDF) [file pone.0226162.s002.pdf]

S2 Table. Antigens selected with the anti-human IgG.

**Identity (blastN)**
HS GRB10 interacting GYF protein 2 (GIGYF2)
HS DExH-box helicase 9 (DHX9)
WD repeat domain

List of antigens identified by the selection of phage display cDNA library from human brain with anti-human IgG.
