# Supplementary material for: Identification of novel non-myelin biomarkers in multiple sclerosis using an improved phage-display approach
Source: PLoS One. 2019 Dec 5;14(12):e0226162. doi: 10.1371/journal.pone.0226162 (PMC6894809; doi:10.1371/journal.pone.0226162)
Supplement: S3 Table — BlastN analysis of 24 positive clones identified by the selection of phage display cDNA library from human brain with pooled and purified IgG from CSF of MS patients. For each clone are indicated: the code of the clone (“Clone”); the clone frequency (“Freq.”) corresponding to the number of clones, over the total clones sequenced, that map to the same antigen; the nucleotide sequence identified by blastN analysis (“Identity-blastN”); the NCBI accession number (“GeneBank No.”) of the identified sequence; the percentage of homology (“% identity”) with the identified sequence; the first and last nucleotide of the identified sequence. (PDF) [file pone.0226162.s003.pdf]

S3 Table. Nucleotide sequence alignments of clones selected with CSF pool.

Clone	Freq.	Identity - blastN	GeneBank No.	% identity	start	end
HB_CSFD3	3/24	HS DEAD-box helicase 24 (DDX24)	NM_020414.3	100	392	744
HB_CSFD12	1/24	HS DEAD-box helicase 24 (DDX24)	NM_020414.3	100	357	744
HB_CSFD8	2/24	HS erythrocyte membrane protein band 4.1 (EPB41)	NM_001166005.1	100	273	436
HB_CSFD6	1/24	HS pleckstrin homology domain containing B2 (PLEKHB2)	NM_017958.2	98	535	751
HB_CSFE12	1/24	HS ST13, Hsp70 interacting protein (ST13)	NM_003932.4	100	554	749
HB_CSFB2	3/24	HS adenylate kinase 5 (AK5)	NM_174858.2	100	1897	1719
HB_CSFE9	3/24	HS CAP-Gly domain containing linker protein 1 (CLIP1)	NM_002956.2	100	3341	3148
HB_CSFA3	1/24	n.a.	n.a			
HB_CSFD4[a]	2/24	n.a.	n.a			
HB_CSFA2[b]	7/24	HS GRB10 interacting GYF protein 2 (GIGYF2)	NM_001103147.1	99	2628	2976

BlastN analysis of 24 positive clones identified by the selection of phage display cDNA library from human brain with pooled and purified IgG from CSF of MS patients. For each clone are indicated: the code of the clone (“Clone”); the clone frequency (“Freq.”) corresponding to the number of clones, over the total clones sequenced, that map to the same antigen; the nucleotide sequence identified by blastN analysis (“Identity-blastN”); the NCBI accession number (“GeneBank No.”) of the identified sequence; the percentage of homology (“% identity”) with the identified sequence; the first and last nucleotide of the identified sequence.

[a]out of frame clones

[b]background clones shared with the anti-human IgG selection
