# Supplementary material for: Identification of novel non-myelin biomarkers in multiple sclerosis using an improved phage-display approach
Source: PLoS One. 2019 Dec 5;14(12):e0226162. doi: 10.1371/journal.pone.0226162 (PMC6894809; doi:10.1371/journal.pone.0226162)
Supplement: S4 Table — BlastP analysis of 24 positive clones identified by the selection of phage display cDNA library from human brain with pooled and purified IgG from CSF of MS patients. For each clone are indicated: the code of the clone (“Clone”); the clone frequency (“Freq.”) corresponding to the number of clones, over the total clones sequenced, that map to the same antigen; the aminoacid sequence identified by blastP analysis using the translation of the clone nucleotide sequence (“Identity-blastN”); the NCBI accession number (“ProtBank No.”) of the identified sequence; the percentage of homology (“% identity”) with the identified sequence; the first and last nucleotide of the identified sequence. The last column reports as the clone can be classified: “ORF” if the clone was in frame and an aminoacid sequence was identified; “mimotope” if the nucleotide and aminoacid sequences identified belong to different gene; “unidentified” if an aminoacid sequence was not identified; “out-of-frame” if the clone was apparently not coding; “background” if the clone was also identified in the selection against only human IgG. (PDF) [file pone.0226162.s004.pdf]

S4 Table. Protein sequence alignments of clones selected with CSF pool.

Clone	Fre q.	Identity - blastP	ProtBank No.	% identity	start	end	classification
HB_CSFD3	3/24	DEAD-box helicase 24	NP_065147.1	100	99	215	ORF
HB_CSFD12	1/24	DEAD-box helicase 24	NP_065147.1	100	87	215	ORF
HB_CSFD8	2/24	Erythrocyte membrane protein band 4.1	NP_001159477.1	100	50	103	ORF
HB_CSFD6	1/24	Pleckstrin homology domain containing B2	NP_001296379.1	97	1	29	ORF
HB_CSFE12	1/24	Hsp70 interacting protein	NP_003923.2	97	30	95	ORF
HB_CSFB2	3/24	Flotillin 2	NP_004466.2	55	28	38	mimotope
HB_CSFE9	3/24	n.a					unidentified
HB_CSFA3	1/24	n.a					unidentified
HB_CSFD4	2/24	n.a					out-of-frame
HB_CSFA2	7/24	GRB10-interacting GYF protein 2	NP_001096618.1	97	761	876	background

BlastP analysis of 24 positive clones identified by the selection of phage display cDNA library from human brain with pooled and purified IgG from CSF of MS patients. For each clone are indicated: the code of the clone (“Clone”); the clone frequency (“Freq.”) corresponding to the number of clones, over the total clones sequenced, that map to the same antigen; the aminoacid sequence identified by blastP analysis using the translation of the clone nucleotide sequence (“Identity-blastN”); the NCBI accession number (“ProtBank No.”) of the identified sequence; the percentage of homology (“% identity”) with the identified sequence; the first and last nucleotide of the identified sequence. The last column reports as the clone can be classified: “ORF” if the clone was in frame and an aminoacid sequence was identified; “mimotope” if the nucleotide and aminoacid sequences identified belong to different gene; “unidentified” if an aminoacid sequence was not identified; “out-of-frame” if the clone was apparently not coding; “background” if the clone was also identified in the selection against only human IgG.
