# Supplementary material for: Identification of novel non-myelin biomarkers in multiple sclerosis using an improved phage-display approach
Source: PLoS One. 2019 Dec 5;14(12):e0226162. doi: 10.1371/journal.pone.0226162 (PMC6894809; doi:10.1371/journal.pone.0226162)
Supplement: S5 Table — BlastN analysis of 42 positive clones identified by the selection of phage display cDNA library from human brain with pooled and purified IgG from sera of MS patients. For the meaning of column heading see the legend of S3 Table. (PDF) [file pone.0226162.s005.pdf]

**S5 Table. Nucleotide sequence alignments of clones selected with sera pool.**

| Clone | Freq. | Identity - blastN | GeneBank No. | % identity | start | end |
|---|---|---|---|---|---|---|
| HB_RRG7 | 1/42 | HS amyloid beta precursor protein (APP) | NM_201413.2 | 95 | 1037 | 1298 |
| HB_RRF1 | 1/42 | HS ATP synthase, H+ transporting, mitochondrial Fo complex subunit E (ATP5I) | NM_007100.3 | 0 | 87 | 240 |
| HB_RRB7 | 1/42 | HS brain expressed X-linked 2 (BEX2) | NM_001168399.1 | 100 | 444 | 700 |
| HB_RRH5 | 1/42 | HS DEAD-box helicase 24 (DDX24) | NM_020414.3 | 100 | 357 | 744 |
| HB_RRC8 | 1/42 | HS DEAD-box helicase 24 (DDX24) | NM_020414.3 | 99 | 392 | 658 |
| HB_RRH11 | 1/42 | HS heterogeneous nuclear ribonucleoprotein U like 2 (HNRNPUL2) | NM_001079559.2 | 100 | 2214 | 2379 |
| HB_RRH2 | 1/42 | HS microtubule associated protein 1B (MAP1B) | NM_005909.4 | 100 | 2189 | 2523 |
| HB_RRB8 | 1/42 | HS adenylate kinase 5 (AK5) | NM_174858.2 | 100 | 1897 | 1719 |
| HB_RRE2 | 1/42 | HS RNA, 28S ribosomal (LOC109910382), ribosomal RNA | NR_146154.1 | 97 | 1861 | 2009 |
| HB_RRC3 | 1/42 | HS cystathionine-beta-synthase (CBS) | NM_000071.2 | 100 | 1622 | 1854 |
| HB_RRA12 | 1/42 | HS hypoxia up-regulated 1 (HYOU1), transcript variant 2 | NM_001130991.2 | 100 | 3998 | 3580 |
| HB_RRA11 | 1/42 | HS RANBP2-like and GRIP domain containing 5 (RGPD5) | NM_005054.2 | 100 | 3487 | 3470 |
| HB_RRC6[a] | 1/42 | HS protein phosphatase 4 regulatory subunit 4 (PPP4R4) | NM_058237.1 | 100 | 3113 | 2891 |
| HB_RRH9[a] | 6/42 | n.a | n.a | | | |
| HB_RRB10[b] | 20/42 | HS GRB10 interacting GYF protein 2 (GIGYF2) | NM_001103147.1 | 99 | 2628 | 2976 |
| HB_RRD12[b] | 3/42 | HS DExH-box helicase 9 (DHX9) | NM_001357.4 | 100 | 3720 | 3975 |

BlastN analysis of 42 positive clones identified by the selection of phage display cDNA library from human brain with pooled and purified IgG from sera of MS patients. For the meaning of column heading see the legend of S3 Table.

[a]out of frame clones

[b]background clones shared with the anti-human IgG selection
