# Supplementary material for: Identification of novel non-myelin biomarkers in multiple sclerosis using an improved phage-display approach
Source: PLoS One. 2019 Dec 5;14(12):e0226162. doi: 10.1371/journal.pone.0226162 (PMC6894809; doi:10.1371/journal.pone.0226162)
Supplement: S6 Table — BlastP analysis of 42 positive clones identified by the selection of phage display cDNA library from human brain with pooled and purified IgG from sera of MS patients. For the meaning of column heading see the legend of S4 Table. (PDF) [file pone.0226162.s006.pdf]

S6 Table. Protein sequence alignments of clones selected with sera pool.

Clone	Freq.	Identity - blastP	ProtBank No.	% identity	start	end	classification
HB_RRG7	1/42	Amyloid beta precursor protein	NP_958816.1	99	299	366	ORF
HB_RRF1	1/42	ATP synthase membrane subunit e	NP_009031.1	97	1	35	ORF
HB_RRB7	1/42	Brain expressed X-linked 2	NP_001161871.1	99	29	114	ORF
HB_RRH5	1/42	DEAD-box helicase 24	NP_065147.1	100	87	215	ORF
HB_RRC8	1/42	DEAD-box helicase 24	NP_065147.1	99	99	186	ORF
HB_RRH11	1/42	Heterogeneous nuclear ribonucleoprotein U like 2	NP_001073027.1	98	663	717	ORF
HB_RRH2	1/42	microtubule-associated protein 1B	NP_005900.2	100	643	753	ORF
HB_RRB8	1/42	Flotillin 2	NP_004466.2	55	28	38	mimotope
HB_RRE2	1/42	Uridine-cytidine kinase 1 like 1	NP_060329.2	50	69	94	mimotope
HB_RRC3	1/42	n.a					unidentified
HB_RRA12	1/42	n.a					unidentified
HB_RRA11	1/42	n.a					unidentified
HB_RRC6	1/42	n.a					out-of-frame
HB_RRH9	6/42	n.a					out-of-frame
HB_RRB10	20/42	GRB10-interacting GYF protein 2	NP_001096618.1	97	761	876	background
HB_RRD12	3/42	ATP-dependent RNA helicase A DHX9	NP_001348.2	100	1183	1267	background

BlastP analysis of 42 positive clones identified by the selection of phage display cDNA library from human brain with pooled and purified IgG from sera of MS patients. For the meaning of column heading see the legend of S4 Table.
