# Supplementary material for: Identification of novel non-myelin biomarkers in multiple sclerosis using an improved phage-display approach
Source: PLoS One. 2019 Dec 5;14(12):e0226162. doi: 10.1371/journal.pone.0226162 (PMC6894809; doi:10.1371/journal.pone.0226162)
Supplement: S7 Table — BlastN analysis of 15 positive clones identified by the selection of phage display cDNA library from human brain with the scFv phage display library from CSF of two RR-MS patients. For the meaning of column heading see the legend of S3 Table. (PDF) [file pone.0226162.s007.pdf]

S7 Table. Nucleotide sequence alignments of clones selected with the scFV library.

Clone	Freq.	Identity - blastN	GeneBank No.	% identity	start	end
HB_scFv-CSFA1	3/15	HS DEAD-box helicase 24 (DDX24)	NM_020414.3	100	357	744
HB_scFv-CSFB5	2/15	HS DEAD-box helicase 24 (DDX24)	NM_020414.3	100	515	786
HB_scFv-CSFA5	1/15	HS DEAD-box helicase 24 (DDX24)	NM_020414.3	100	392	744
HB_scFv-CSFB7	1/15	HS transcription elongation factor A like 4 (TCEAL4)	NM_024863.5	100	591	743
HB_scFv-CSFE5	1/15	HS transcription elongation regulator 1 (TCERG1)	NM_006706.3	100	2085	2418
HB_scFv-CSFB6	2/15	HS adenylate kinase 5 (AK5)	NM_174858.2	100	1897	1719
HB_scFv-CSFA8	2/15	HS Fas apoptotic inhibitory molecule 2 (FAIM2)	NM_012306.3	99	3655	3585
HB_scFv-CSFG6	1/15	HS cathepsin C (CTSC)	NM_148170.4	93	4132	4078
HB_scFv-CSFG7	1/15	HS vesicle associated membrane protein 1 (VAMP1)	NM_014231.4	99	2402	2128
HB_scFv-CSFF4[a]	1/15	HS RNA, 28S ribosomal (LOC109910382), ribosomal RNA	NR_146154.1	100	3933	3734

BlastN analysis of 15 positive clones identified by the selection of phage display cDNA library from human brain with the scFv phage display library from CSF of two RR-MS patients. For the meaning of column heading see the legend of S3 Table.

[a]out of frame clones
