# Supplementary material for: Identification of novel non-myelin biomarkers in multiple sclerosis using an improved phage-display approach
Source: PLoS One. 2019 Dec 5;14(12):e0226162. doi: 10.1371/journal.pone.0226162 (PMC6894809; doi:10.1371/journal.pone.0226162)
Supplement: S8 Table — BlastP analysis of 15 positive clones identified by the selection of phage display cDNA library from human brain with the scFv phage display library from CSF of two RR-MS patients. For the meaning of column heading see the legend of S4 Table. (PDF) [file pone.0226162.s008.pdf]

S8 Table. Protein sequence alignments of clones selected with the scFv library.

Clone	Freq.	Identity - blastP	ProtBank No.	% identity	start	end	classification
HB_scFv-CSFA1	3/15	DEAD-box helicase 24	NP_065147.1	100	87	215	ORF
HB_scFv-CSFB5	2/15	DEAD-box helicase 24	NP_065147.1	100	140	229	ORF
HB_scFv-CSFA5	1/15	DEAD-box helicase 24	NP_065147.1	100	99	215	ORF
HB_scFv-CSFB7	1/15	Transcription elongation factor A like 4	NP_001006936.1	100	98	148	ORF
HB_scFv-CSFE5	1/15	Transcription elongation regulator 1	XP_004042807.1	99	683	793	ORF
HB_scFv-CSFB6	2/15	Flotillin 2	NP_004466.2	55	28	38	mimotope
HB_scFv-CSFA8	2/15	Phosphatidyl inositol-4-phosphate 5-kinase type 1 alpha	XP_006711630.1	50	450	465	mimotope
HB_scFv-CSFG6	1/15	n.a					unidentified
HB_scFv-CSFG7	1/15	n.a					unidentified
HB_scFv-CSFF4	1/15	n.a					out-of-frame

BlastP analysis of 15 positive clones identified by the selection of phage display cDNA library from human brain with the scFv phage display library from CSF of two RR-MS patients. For the meaning of column heading see the legend of S4 Table.
