# Supplementary material for: Identification of novel non-myelin biomarkers in multiple sclerosis using an improved phage-display approach
Source: PLoS One. 2019 Dec 5;14(12):e0226162. doi: 10.1371/journal.pone.0226162 (PMC6894809; doi:10.1371/journal.pone.0226162)
Supplement: S1 Fig — The diagnostic value of DDX24 and TCERG1 was further investigated testing the reactivity of some sera samples (30 MS from RR-MS samples utilized in the selections and 38 OND with a mean age of 62 and a ratio of female/male of 14/24) against synthetic peptides pTCERG1 (A) and pDDX24 (C) by an ELISA assay. The synthetic peptides named pDDX24 (aa SQSTAAKVPKKAKTWIPEVHD) and pTCERG1 (aa AAKHAKDSRFKAIEKMKDRE) are included in the aminoacidic portion of antigens recognized in the selections. Unpaired t-test has been used in A and C (**** p< 0.0001). A significantly higher reactivity of MS patients against pDDX24 and pTCERG1 compared to the control group was observed (S1A and S1C Fig). The data of the Receiver operating characteristic (ROC) curve analysis for the pDDX24 and pTCERG1 ELISA are showed near the graph (S1B and S1D Fig). For pDDX4 at O.D. cut off of 0.0765 the sensitivity for discriminating patients with and without MS is of 53.33% (95% confidence interval 34.33–71.66) and specificity of 89.74% (95% confidence interval 75.78–97.13) with a prevalence weighted likelihood positive ratio (LR+) of 5.2 for the diagnosis of MS. For pTCERG1 at O.D. cut-off of 0.055 the test showed a sensitivity of 73.33% (95% confidence interval 54.11–87.72) and a specificity of 81.58% (95% confidence interval 65.67–92.26) with a LR+ of 3.98. (PDF) [file pone.0226162.s009.pdf]

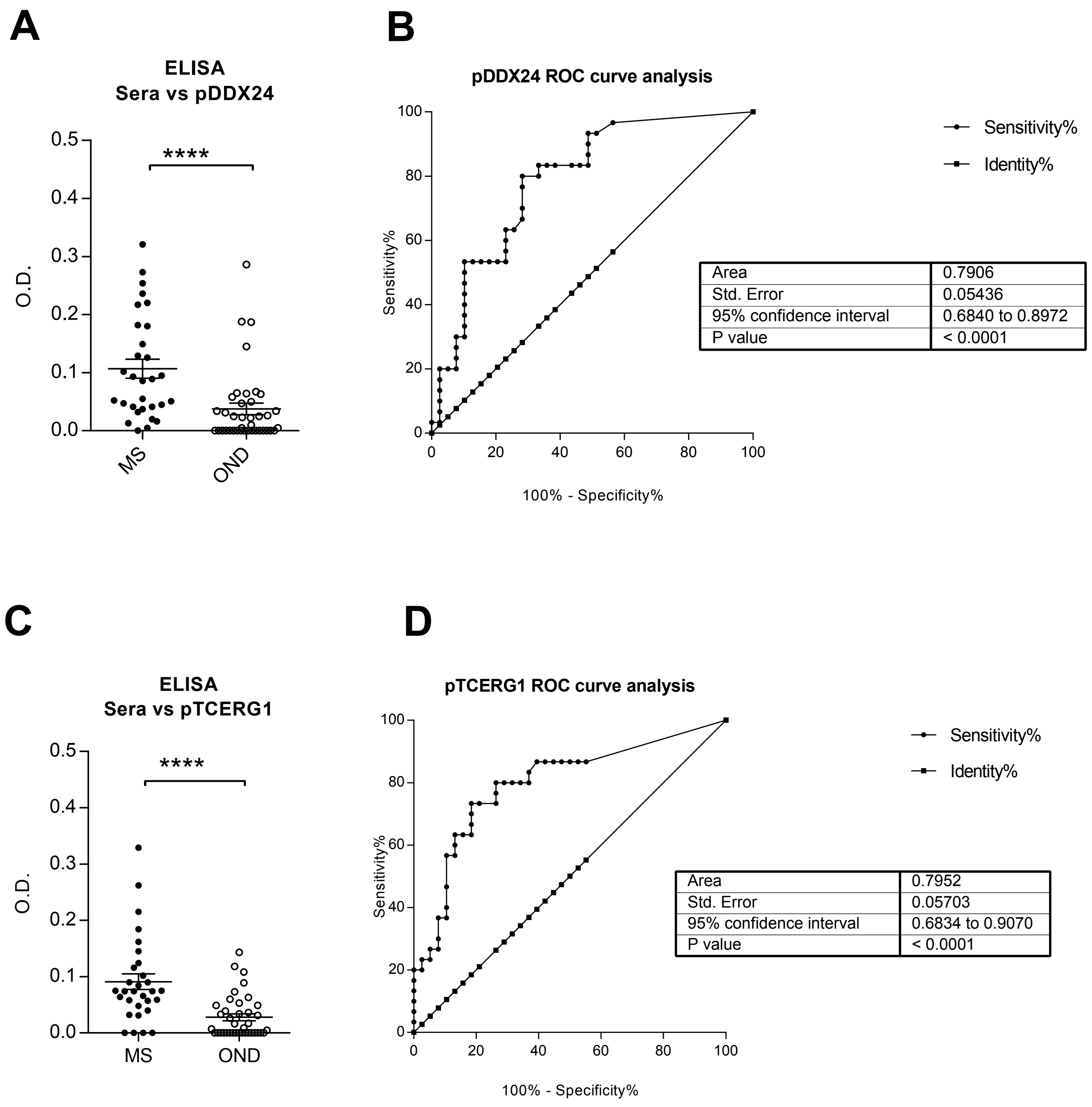

S1 Figure. Evaluation of the diagnostic value of pDDX24 and pTCERG1 in the prediction of MS.
The diagnostic value of DDX24 and TCERG1 was further investigated testing the reactivity of some sera samples (30 MS from RR-MS samples utilized in the selections and 38 OND with a mean age of 62 and a ratio of female/male of 14/24) against synthetic peptides pTCERG1 (A) and pDDX24 (C) by an ELISA assay. The synthetic peptides named pDDX24 (aa SQSTAAKVPKKAKTWIPEVHD) and pTCERG1 (aa AAKHAKDSRFKAIEKMKDRE) are included in the aminoacidic portion of antigens recognized in the selections. Unpaired t-test has been used in A and C (**** p< 0.0001). A significantly higher reactivity of MS patients against pDDX24 and pTCERG1 compared to the control group was observed (Fig S1 A, C).
The data of the Receiver operating characteristic (ROC) curve analysis for the pDDX24 and pTCERG1 ELISA are showed near the graph (Fig S1 B, D). For pDDX4 at O.D. cut off of 0.0765 the sensitivity for discriminating patients with and without MS is of 53.33% (95% confidence interval 34.33-71.66) and specificity of 89.74% (95% confidence interval 75.78-97.13) with a prevalence weighted likelihood positive ratio (LR+) of 5.2 for the diagnosis of MS. For pTCERG1 at O.D. cut-off of 0.055 the test showed a sensitivity of 73.33% (95% confidence interval 54.11-87.72) and a specificity of 81.58% (95% confidence interval 65.67-92.26) with a LR+ of 3.98.
