# Supplementary material for: Identification of novel non-myelin biomarkers in multiple sclerosis using an improved phage-display approach
Source: PLoS One. 2019 Dec 5;14(12):e0226162. doi: 10.1371/journal.pone.0226162 (PMC6894809; doi:10.1371/journal.pone.0226162)
Supplement: S2 Fig — Serum response against synthetic peptides pTCERG1 and pDDX24 in MS (n = 30) and OND (n = 38) patients measured by ELISA (S1 Fig). Individual biological replicates are shown. Dotted lines represent the cut-off with the highest Youden’s index calculated for each antigen. Considering positive only the double positive samples the test showed a sensitivity of 43.33% and a specificity of 97.37%; the PPV and FPR were respectively 92.86% and 2.63% with a LR+ of 16.47. (PDF) [file pone.0226162.s010.pdf]

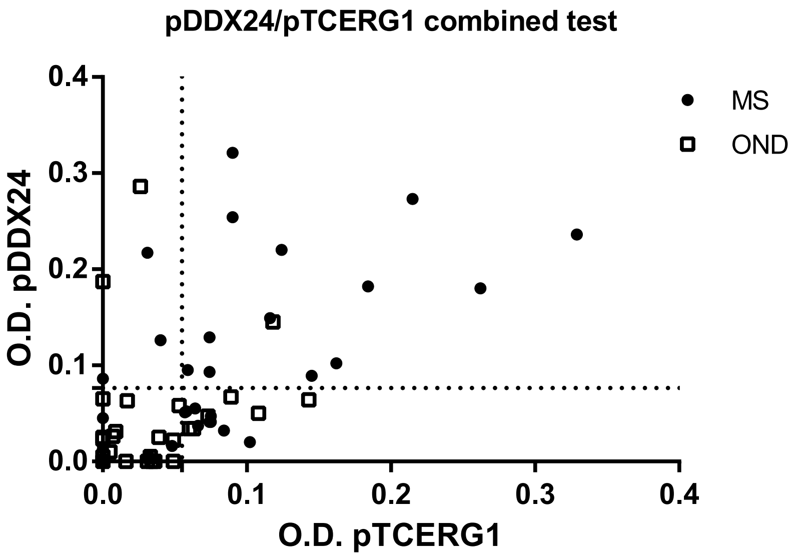

S2 Fig. Evaluation of the diagnostic value of pDDX24/pTCERG1 combined test in the prediction of MS.

Serum response against synthetic peptides pTCERG1 and pDDX24 in MS (n=30) and OND (n=38) patients measured by ELISA (S1 Fig). Individual biological replicates are shown. Dotted lines represent the cut-off with the highest Youden's index calculated for each antigen.

Considering positive only the double positive samples the test showed a sensitivity of 43.33% and a specificity of 97.37%; the PPV and FPR were respectively 92.86% and 2.63% with a LR+ of 16.47.
