# Supplementary material for: Identification of novel non-myelin biomarkers in multiple sclerosis using an improved phage-display approach
Source: PLoS One. 2019 Dec 5;14(12):e0226162. doi: 10.1371/journal.pone.0226162 (PMC6894809; doi:10.1371/journal.pone.0226162)
Supplement: Attachment — Submitted filename: Rebuttal letter PONE-D-19-17789.pdf [file pone.0226162.s011.pdf]

MS # PONE-D-19-17789

Responses to questions of the reviewers

Reviewer #1

1) Line 118, phrase starting with "Relatively to CSF…" double check the English.

The sentence has been corrected.

Old sentence
Relatively to the CSFs, were pooled samples of 7 untreated RR MS patients (EDSS 0-3.5) and 4 clinically isolated syndrome (CIS) patients (EDSS 0-2).

New sentence
For the selection against CSF samples, were pooled samples of 7 untreated RR MS patients (EDSS 0-3.5) and 4 clinically isolated syndrome (CIS) patients (EDSS 0-2).

2) Table 1. I think the term "range" should be removed for the "mean age" column, after all only the mean age is shown in the table.

The term "range" has been removed for the "**mean age**" column, as correctly suggested.

3) Line 143. Is it correct that only 1 picogram of poly(A)+ RNA and 2.5 pg of random primers were used for the generation of the library? Similarly, in line 159 the authors describe the use of only 40 pL of cells for electrocompetent cells transformation.

The use of "p" for pg and pL at lines 143 and 159 was a typographical error and it has been corrected in µg and µL.

4) Line 352. How many clones were screened to identify the three "background" antigens? I think it is important to state the number to give more significance to the fact that only three clones were identified. In order to reduce binding background, why the authors did not considered to use anti-IgG antibody to pre-clear the phage population before the selection?

To identify the three "background" antigens 40 clones have been screened. As suggested by reviewer, this data has been included in the text.
About the suggestion to preclear the phage library, it is necessary to specify that the construction of the library from human brain was a very complex procedure, in particular for the initial quantity of RNA available and we preferred to use the library directly for the selection. A pre-clearing step has been evaluated and will be included in the future improvement of the protocol. Nevertheless the results from the background analysis were convincing.

Old sentence
After three rounds of selections on the HB library, only three antigens specifically enriched by the anti-human antibody were observed (S2 Table).

New sentence
After three rounds of selections on the HB library, **40 clones were screened and** only three antigens specifically enriched by the anti-human antibody were observed (S2 Table).

5) Line 415. What do the authors mean with a scFv antibody library diversity of 73%? How did they estimate the diversity? If 27% of the clones were present more than once in a very small library of $2x10^4$ clones I questioned the quality of the library itself.

We thank the reviewer for the comment and we agree that a better explanation should be given. We express the scFv antibody library diversity as percentage of unique VL fragment in our library, which we evaluated by PCR followed by enzymatic fingerprinting (line 178 Material and Methods) of random clones.
It should be noted that since the B cell present in the CSF of MS patients are a very oligoclonal population, the fact that 27% of the clones would share the same VL fragment is expected and in accordance with previously described data (Owens et al. J Immunol 2003).
We have revised the related sentence.

Old sentence
In particular, a single chain (scFv) phage display library of 2x10^{4} independent clones and a diversity of 73% was generated starting from a pool of B cells purified from the liquor of two MS patients and used to select the HB phage library as described in methods.

New sentence
In particular, a single chain (scFv) phage display library of 2x10^{4} independent clones ~~**and a diversity of 73%**~~ was generated starting from a pool of B cells purified from the liquor of two MS patients. **The library diversity was evaluated as usage of unique VL gene per clones and measured as described in Methods. An estimated value of 73% was calculated. The scFv library was** used to select the HB phage library as described in Methods.

6) Supplementary Table S7. In the legend and in the text, it is mentioned that 15 positive clones were identified, while in the table the clone frequencies are reported to be fractions of 17 clones (i.e. 1/17).

The number 17 in Supplementary Table S7 was a typographical error and it has been corrected in **15**.

7) Figure 2. Figure Legend. In the figure for DDX24 there are * indicating the statistical significance, while for TCERG1 there are **, but in the figure legend the p value is indicated for ** and *** stars.***

The correct value is $p < 0.0001$, correspondent to four asterisks, and it has been corrected in the legend.

8) Line 497. The authors state that "a bigger cohort is needed to calculate this value with a better precision". Since the described experiment involved the use of recombinant purified proteins, instead of protein fragments displayed on the surface of the phage, the authors should repeat the experiment adding all the other sera utilized in the previous part of the study, where the proteins were displayed on phage, to increase the cohort size and better validate their findings.

We agree with the suggestion of the reviewer but a clarification about the guide lines on the use of multiple sclerosis patients samples could be useful. Using MS sera in diagnostic assays, as ELISA tests, it is preferred to employ fresh sample or unfrozen one time at most. For this reason, we could not use all the sera utilized in the selection also in the validation tests; further in some cases the available quantity was very low.
In any case, as suggested, we have performed another ELISA analysis with a bigger cohort of samples utilized in the selections; since, in the meantime, we have tested some synthetic peptides correspondent to the recognized portion of the identified antigens, we preferred to use this type of antigen in the assay, in particular for DDX24 antigen for which it is necessary to denature the protein.

Consequently we have included a Figure that describes the ELISA assay as Supplementary material and we have revised the sentence about the combined test using the recombinant proteins.

Revision in the section Materials and Methods

Line 307 old sentence
96-well flat-bottomed Reacti-Bind™ Pierce plates were coated overnight at RT with 10 µg/ml of recombinant protein in PBS per well, an equivalent number of wells were coated with PBS only (background).

Line 307 new sentence

96-well flat-bottomed Reacti-Bind™ Pierce plates were coated overnight at RT with **1 μg of recombinant protein or 2 μg of synthetic peptide** in PBS per well, an equivalent number of wells were coated with PBS only (background).

Supplementary figures added

S1 Figure. Evaluation of the diagnostic value of pDDX24 and pTCERG1 in the prediction of MS.

The diagnostic value of DDX24 and TCERG1 was further investigated testing the reactivity of some sera samples (30 MS from RR-MS samples utilized in the selections and 38 OND with a mean age of 62 and a ratio of female/male of 14/24) against synthetic peptides pTCERG1 (A) and pDDX24 (C) by an ELISA assay. The synthetic peptides named pDDX24 (aa SQSTAAKVPKKAKTWIPEVHD) and pTCERG1 (aa AAKHAKDSRFKAIEKMKDRE) are included in the aminoacidic portion of antigens recognized in the selections. Unpaired t-test has been used in A and C (** p< 0.0001). A significantly higher reactivity of MS patients against pDDX24 and pTCERG1 compared to the control group was observed (Fig S1 A, C).**

The data of the Receiver operating characteristic (ROC) curve analysis for the pDDX24 and pTCERG1 ELISA are showed near the graph (Fig S1 B, D). For pDDX4 at O.D. cut off of 0.0765 the sensitivity for discriminating patients with and without MS is of 53.33% (95% confidence interval 34.33-71.66) and specificity of 89.74% (95% confidence interval 75.78-97.13) with a prevalence weighted likelihood positive ratio (LR+) of 5.2 for the diagnosis of MS. For pTCERG1 at O.D. cut-off of 0.055 the test showed a sensitivity of 73.33% (95% confidence interval 54.11-87.72) and a specificity of 81.58% (95% confidence interval 65.67-92.26) with a LR+ of 3.98.

S2 Figure. Evaluation of the diagnostic value of pDDX24/pTCERG1 combined test in the prediction of MS.

Serum response against synthetic peptides pTCERG1 and pDDX24 in MS (n=30) and OND (n=38) patients measured by ELISA (S1 Fig). Individual biological replicates are shown. Dotted lines represent the cut-off with the highest Youden's index calculated for each antigens.

Considering positive only the double positive samples the test showed a sensitivity of 43.33% and a specificity of 97.37%; the PPV and FPR were respectively 92.86% and 2.63% with a LR+ of 16.47.

Line 497 old sentence

We then assessed the possibility to improve the specificity and sensitivity using both antigens in a combined test for the diagnosis of MS (Fig 4). In this case, considering positive only the double positive samples the test showed a sensitivity of 72,2% and a specificity of 100%. Since for this condition the PPV is 100% and the FPR tends to infinite, a bigger cohort is needed to calculate this value with a better precision. The low sensitivity could be dependent on the heterogeneity of immune response in MS patients.

Line 497 new sentence

We then assessed the possibility to improve the specificity and sensitivity using both antigens in a combined test for the diagnosis of MS (Fig 4). In this case, considering positive only the double positive samples the test showed a sensitivity of 72,2% and a specificity of 100%; **for this condition the PPV is 100% and the LR+ tends to infinite. We tested also the reactivity of synthetic peptides obtained from the sequence of DDX24 and TCERG1 against a bigger cohort of sera samples including sera from MS patients utilized in the selections (see S1 Fig for the data). Combining these results as before, the sensitivity was of 43.33% and the specificity of 97.37%; for these values the PPV is 92.86% and LR+ is 16.47. In both analysis using combined test of two antigens, the sensitivity was low and this could be dependent on the heterogeneity of immune response in MS patients; however the specificity was higher respect to use a single antigen. It should be noted that TCERG1 gave better result to discriminate MS and OND samples as recombinant protein whereas DDX24 as synthetic peptide.**

9) Line 550. The authors list the benefits of using recombinant scFv for the isolation of potential autoantigens. I found their conclusions not too convincing. It is known that, when phage selections are performed, the enrichment ratio does not necessarily reflect the quality of a selection. Even in the present work, although the selection performed by using scFv gave a 160-fold enrichment, only 15 out of 94 clones were positive during the ELISA screening (~16%) while 48% of the clones were positive when purified antibodies from CSF were used, with only a 10 fold enrichment. Moreover, the identification of the background clones was obtained selecting the cDNA library against an anti-human-IgG antibody, so the presence of no background when phage scFv directly bound on plastic

are used for the selection is not surprise. A selection on an unrelated phage-scFv should have been used to assess the background of this selection strategy.

We agree with the reviewer that the fold enrichment cannot be correlated with a better selection; however, we think that our observations support our statement on the advantage to perform selection with the scFv library and a better clarification on the classification of clones (S4, S6 and S8 tables) might help to explain our conclusions. All the clones classified as ORF, mimotope or “unidentified antigens” correspond to “in-frame” clones being their translated sequence without stop codons. Of consequence, although it is true that the CSF selection lead to 48% of the clones positive in Phage-ELISA, of these only 62,5% (15/24) were clones with an “in-frame” insert while the others were background clones (S4 table). In contrast, although the selection with scFv lead to have only 16% of positive clones in phage-ELISA, of these 93% (14/15) were “in-frame” clones (S8 table). Moreover, one of the antigens (TCERG1), able to discriminate MS from OND patients, has been selected only with scFv library indicating that the use of this system could overcome limitation in the use of Ig directly from the CSF where the low concentration of IgG in the CSF of MS patients or the low abundance of some specific antibody could be a limiting factor in the selection of specific autoantigens.

To better explain these observations and accepting the suggestion of the reviewer, we revised the sentence where we described the classification of the clones and we modified the sentence about the advantages of selection with scFv.

Line 377 – old sentence

Apart from background or out-of-frame clones, the clones matching a nucleotide, but not an aminoacid, sequence, were classified as "unidentified antigens".

Line 377 -new sentence

Apart from background or out-of-frame clones, the clones matching a nucleotide, but not aminoacid, sequence, were classified as "unidentified antigens", **even if they correspond to an “in-frame” sequences without stop codons.**

Line 550 - old sentence

Selection with the scFv library, in fact, showed the best enrichment ratio (160-fold compare to 5.4 or 10-fold for serum IgG and CSF IgG respectively), the lowest percentage of background clones and a good specificity isolating a restricted number of molecular targets with relative high frequency.

New sentence

Selection with the scFv library, in fact, showed the **highest percentage (14/15 corresponding to a percentage of 93%) of “in-frame” clones identified (S8 table)** and a good specificity isolating a restricted number of molecular targets with relative high frequency.

Reviewer #2

1) To generate the scFv library the authors selected two drug-naïve RR-MS patients. Where the RR-MS patients of the validation cohort without any treatment as well? Please specify in the text

Also the RR-MS patients of the validation cohort were without any treatment. This has been specified as suggested.

Added sentence at the end of paragraph Serum and CSF samples

All samples used in ELISA for validation tests were from untreated patients.

2) A figure showing a schematic overview of the generation of library/screening would be helpful for readers

A figure showing a schematic overview of the generation of library and screening has been included as suggested by the reviewer and consequently all the figures has been renumbered. A sentence in the section Results has been included.

Line 359 old sentence
To identify the epitopes recognized by these antibodies we selected the HB phage library with a IgG pool of 11 CSFs collected from MS patients with a primary diagnosis of the disease (7 RR and 4 CIS) as described in Methods.

Line 359 new sentence
To identify the epitopes recognized by these antibodies **we performed three different biopanning of the HB phage library as summarized in the schematic overview of Fig 1. Initially** we selected the HB phage library with a IgG pool of 11 CSFs collected from MS patients with a primary diagnosis of the disease (7 RR and 4 CIS) as described in Methods.

Figure 1 legend
Schematic overview of the phage display selection approach for the identification of candidate autoantigens in multiple sclerosis.

3) Abbreviation and acronyms should be specified the first time they appear in the text. I found the full name of the two new autoantigens DDX24 (DEAD-Box Helicase 24) and TCERG1 (Transcription Elongation Regulator 1) at the end of the discussion. Please correct all along the text for all abbreviations.

The abbreviations were checked throughout the entire text. The full name of the two new autoantigens DDX24 and TCERG were correctly specified the first time they appear, as correctly requested.

4) lines 593-608: the authors should rephrase this part, because in this study they are not demonstrating any pathogenetic mechanism about the effect of these autoantibodies. They might change the word "propose" with "can speculate"

We agree with the reviewer and the sentence has been changed as suggested.

Old sentence
We therefore propose a mechanism by which autoantibodies against TCERG1 and DDX24 might exert a pathogenetic role in MS inducing or sustaining neurodegeneration by impairing the functions of their target proteins either inducing misplacement or blocking their activity.

New sentence
We therefore **can speculate** a mechanism by which autoantibodies against TCERG1 and DDX24 might exert a pathogenetic role in MS inducing or sustaining neurodegeneration by impairing the functions of their target proteins either inducing misplacement or blocking their activity.

5) In Figure legend 1 the p value is missing

The p value in Figure legend 1 has been indicated.

6) line 781: number 2 is repeated twice

The typographical error has been corrected.

Note: we have corrected all the cross reference to the section Material and Methods.
