# Supplementary material for: Identification of novel non-myelin biomarkers in multiple sclerosis using an improved phage-display approach
Source: PLoS One. 2019 Dec 5;14(12):e0226162. doi: 10.1371/journal.pone.0226162 (PMC6894809; doi:10.1371/journal.pone.0226162)
Supplement: Attachment — Submitted filename: Rebuttal letter2 PONE-D-19-17789.pdf [file pone.0226162.s012.pdf]

MS # PONE-D-19-17789

Responses to reviewers

Reviewer #1

- **Line 120** in the revisited manuscript:
"For the selection against CSF samples, were pooled samples of 7 untreated RR MS patients (EDSS 0-3.5) and 4 clinically isolated syndrome (CIS) patients (EDSS 0-2)"
I am pretty confindent that the correct English version of such sentence should be:
"Samples from 7 untreated RR MS patients (EDSS 0-3.5) and 4 clinically isolated syndrome (CIS) patients (EDSS 0-2) were pooled for the selection against CSF samples".

We agree with the reviewer and the sentence has been corrected.

-**Line 431**. I think it is worth to mention the citation of Owen et al. 2003 to briefly explain the oligoclonal nature of the antibodies obtained from CSF of MS patients.

The bibliographic reference has been cited and the following sentence has been added.

Old Sentence

An estimated value of 73% was calculated. The scFv library was used to select the HB phage library as described in Methods.

New sentence

An estimated value of 73% was calculated. **It should be noted that, since the B cell present in the CSF of MS patients are a very oligoclonal population, the fact that 27% of the clones would share the same VL fragment is expected and in accordance with previously described data [27].** The scFv library was used to select the HB phage library as described in Methods.

The references of the citations has been revised accordingly.
